# Best medical treatment vs endovascular repair for uncomplicated acute type B aortic dissection: a comparative study

**DOI:** 10.20452/wiitm.2025.17972

**Published:** 2025-07-24

**Authors:** Fu ‑Kang Yuan, Hong ‑Yi Yang, Yu‑Fei Fu, Zhong Tian, Hua‑Wei Zhuo, Ming‑Bao Chen

**Affiliations:** Department of General Surgery Xuzhou Central Hospital Xuzhou Clinical School of Xuzhou Medical University, Xuzhou China; Key Laboratory for Biotechnology on Medicinal Plants of Jiangsu Province, School of Life Science Jiangsu Normal University Xuzhou China; Department of Radiology Xuzhou Central Hospital Xuzhou China; Department of Cardiovascular Surgery Xuzhou Central Hospital Xuzhou China

**Keywords:** aortic dissection, endovascular, medical treatment, stent, type B

## Abstract

**INTRODUCTION:**

Both best medical treatment (BMT) and endovascular repair (ER) are viable treatment strategies in uncomplicated acute type B aortic dissection (TBAD). However, long-term outcomes of these 2 approaches remain a topic of debate.

**AIM:**

This study was developed to compare the clinical efficacy, short-term outcomes, and long-term results of ER and BMT in the management of uncomplicated TBAD.

**MATERIALS AND METHODS:**

This retrospective, single-center study included consecutive individuals diagnosed with uncomplicated TBAD who underwent ER or BMT between January 2019 and August 2024. Relative outcomes for these 2 treatment approaches were compared.

**RESULT:**

In total, 165 and 148 patients who respectively received ER and BMT were enrolled in the analysis. Relative to BMT, ER significantly increased the thrombosed / obliterated false lumen rate (81.8% vs 16.2%, respectively; *P* = 0.001), reduced the rupture rate (1.8% vs 12.2%, respectively; *P* = 0.001), and decreased late mortality (4.8% vs 14.2%, respectively; *P* = 0.004). The rates of retrograde type A dissection, organ failure, and early mortality in the BMT and ER groups were similar (1.8% vs 3.4%; *P* = 0.48; 0.6% vs 2%; *P* = 0.35; 0.6% vs 4.1%; *P* = 0.06, respectively). In the ER group, the overall survival rates at 1, 3, and 5 years were 98.8%, 96.5%, and 94.6%, respectively, while the BMT group exhibited corresponding survival rates of 94.5%, 91.5%, and 84.7%.

**CONCLUSION:**

In comparison with BMT, ER significantly reduces rupture rate and enhances thrombosed / obliterated false lumen rate, thereby improving long-term prognosis.

## INTRODUCTION

Acute type B aortic dissection (TBAD) is a critical medical condition.^1^;^2^;^3^ Based on clinical presentation, TBAD cases are classified as either complicated or uncomplicated. Complicated TBAD is characterized by refractory pain, malperfusion syndromes, rapid aortic expansion, or rupture at the onset or in the course of hospitalization, whereas uncomplicated TBAD lacks these high-risk features. As such, the former needs to be managed via endovascular repair (ER) in most cases, whereas the latter can be managed with either ER or best medical treatment (BMT).^4^

While short-term outcomes of ER and BMT are comparable, the long-term benefits of each approach remain a subject of controversy.^1^;^2^;^3^ Some meta-analyses suggest that ER offers a higher thrombosed / obliterated false lumen rate, a lower rupture rate, and reduced aortic-related mortality, as compared with BMT.^1^;^3^ However, a meta-analysis^2^ has reported no significant differences in 4- or 5-year survival between the 2 treatments.

## AIM

This study aimed to evaluate and compare the clinical efficacy as well as short-and long-term results of ER and BMT in treating uncomplicated TBAD.

## MATERIALS AND METHODS

### Study design

This retrospective, single-center study was approved by the Ethics Committee of Xuzhou Central Hospital (XZXY-LJ-20151225-0890, with a waiver for written informed consent. Consecutive patients diagnosed with uncomplicated TBAD who underwent ER or BMT between January 2019 and August 2024 were included. Inclusion criteria involved uncomplicated TBAD, age between 18 and 80 years, and symptom onset less than 14 days before treatment. Exclusion criteria comprised maximum thoracic aorta transverse diameter over 55 mm or liver, kidney, or lung dysfunction.

TBAD was diagnosed based on clinical symptoms, symptom onset timing, and computed tomography angiography (CTA) findings. Anatomical characteristics, false lumen thrombosis status, and dissection extent were evaluated prior to treatment.

### Treatments

Treatment decisions were made through a shared decision-making process involving the patient and their family with full informed consent for treatment decisions. For the patients receiving ER, stent-graft size was selected based on the aortic diameter and the distance from the left subclavian artery (LSA) to the primary entry tear. When this distance was below 2 cm, angiography was performed to assess vertebrobasilar circulation before determining LSA coverage. Coverage of the LSA without reconstruction was performed if there was sufficient collateral circulation; otherwise, LSA reconstruction was carried out. Coverage of the left common carotid artery (LCCA) was not allowed. When necessary, LSA or LCCA reconstruction was conducted using the endovascular chimney technique to ensure an adequate proximal landing zone.

All patients in both groups received antihypertensive therapy (calcium channel blockers, nitroglycerine, β-blockers, or combinations thereof) if their systolic blood pressure (SBP) exceeded 120 mm Hg on presentation. The initial goal was to reduce SBP to 100–120 mm Hg and alleviate pain when providing BMT. Persistent chest pain was managed with intravenous non-narcotic (buprenorphine hydrochloride) or narcotic (morphine hydrochloride) analgesics.

### Assessment

The primary end point of the study was the rate of false lumen thrombosis or obliteration. Secondary end points included complications, early mortality (within 30 days of treatment), late mortality (beyond 30 days after treatment), and aorta-related mortality. The overall survival (OS) was calculated from the point of treatment to patient death (for any reason) or last follow-up (February 2025).

Follow-up assessments were conducted at 3 and 6 months, then annually. Each follow-up visit included a clinical evaluation and CTA imaging. Patients experiencing symptoms could seek medical attention at any time. The end points of follow-up included patient death, loss to follow-up, and as of February 2025. The patients lost to follow-up were marked as alive if thet were alive at the last follow-up .

### Statistical analysis

Continuous variables were expressed as mean (SD) when normally distributed and were compared with the independent-sample *t* tests. Skewed data were presented as median (interquartile range [IQR]) and compared with the Mann–Whitney tests. Categorical variables were analyzed using the χ² or the Fisher exact test. Survival outcomes were evaluated using the Kaplan–Meier curves and log-rank tests. A *P* value below 0.05 was deemed significant. Statistical analyses were conducted with SPSS Statistics software, vesrion 16.0 (IBM, Armonk, New York, United States).

## RESULTS

### Patients

**Figure 1:**
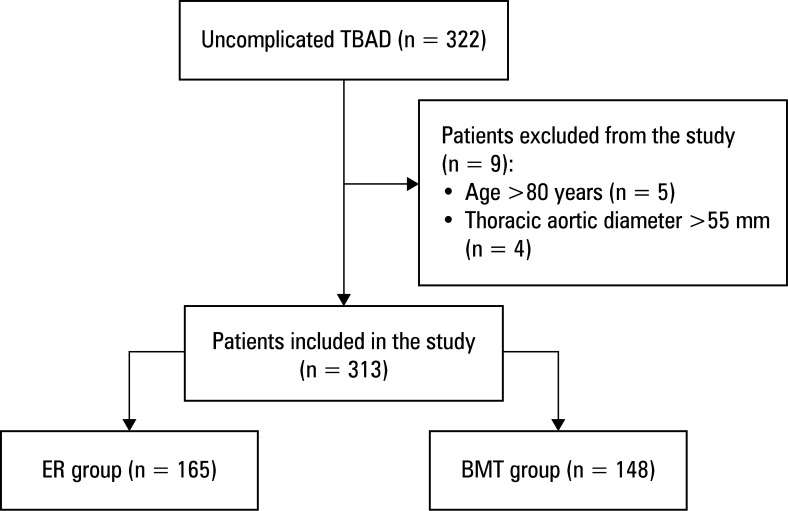
Study flowchart

Abbreviations: BMT, best medical treatment; ER, endovascular repair; TBAD, type B acute aortic dissection

Overall, 165 patients who underwent ER and 148 who received BMT were included in the study Figure 1. Baseline characteristics were comparable between these groups, as outlined in Table 1. Among the ER patients, 11 underwent chimney stenting for LSA (n = 10) or LCCA (n = 1). Median (IQR) follow-up was 56 (38–60) months, with complete follow-up of all patients.

**Table 1:** Baseline patient data

Variable	ER group (n = 165)	BMT group (n = 148)	*P* value
Age, y, mean (SD)	59.5 (13.9)	60.4 (13.6)	0.57
Sex			
Men	142 (86.1)	121 (81.8)	0.3
Women	23 (13.9)	27 (18.2)	
Comorbidities			
Hypertension	72 (43.6)	60 (40.5)	0.58
Diabetes	16 (9.7)	15 (10.1)	0.9
Coronary artery disease	21 (12.7)	17 (11.5)	0.74
Extent of dissection			
Thoracic aorta	22 (13.3)	25 (16.9)	0.38
Extended to abdominal aorta	143 (86.7)	123 (83.1)	
False lumen patency			
Patent	95 (57.6)	82 (55.4)	0.7
Partial thrombosis	70 (42.4)	66 (44.6)	

Data are presented as numbers (percentages) unless indicated otherwise.

Abbreviations: see Figure 1

### Thrombosed / obliterated false lumen incidence

The rate of thrombosed / obliterated false lumen was significantly higher in the ER group (81.8%) than the BMT group (16.2%; *P* = 0.001; Table 2).

**Table 2:** Treatment‑related outcomes

Outcome	ER group (n = 165)	BMT group (n = 148)	*P* value
Thrombosed / obliterated false lumen	135 (81.8)	24 (16.2)	0.001
Complications			
Type I endoleak	10 (6.1)	–	–
Rupture	3 (1.8)	18 (12.2)	0.001
Retrograde type A dissection	3 (1.8)	5 (3.4)	0.48
Organ failure	1 (0.6)	3 (2)	0.35
Early mortality	1 (0.6)	6 (4)	0.06
Late mortality	8 (4.8)	21 (14.2)	0.004
Aortic-related mortality	3 (1.8)	18 (12.2)	0.001

Data are presented as numbers (percentages).

Abbreviations: see Figure 1

### Complications

In the ER group, type I endoleak was detected in 10 participants (6%) immediately after ER. These patients were managed with observation rather than intervention, and at last follow-up, no additional complications had been reported.

TBAD rupture occurred in 3 patients (1.8%) in the ER group and 18 individuals (12.2%) in the BMT group (*P* = 0.001; Table 2). The incidence of retrograde type A dissection (RTAD) (1.8% in the ER group vs 3.4% in the BMT group) and organ failure (0.6% in the ER group vs 2% in the BMT group) was similar between the groups (*P *= 0.48 and *P* = 0.35, respectively). In the ER group, all 3 cases of rupture were attributed to RTAD. In the BMT group, rupture was caused by RTAD in 4 patients.

### Early mortality

In the ER group, 1 patient died due to multiorgan failure. In the BMT group, 6 individuals died: 3 due to multiorgan failure and 3 due to rupture. The difference in early mortality between the groups was insignificant Table 2.

### Late mortality

Eight patients in the ER group suffered from late mortality, attributed to rupture (n = 3), pulmonary infection (n = 3), and cancer (n = 2). In contrast, 21 patients in the BMT group experienced late mortality, with causes including rupture (n = 15), cardiac disease (n = 5), and cancer (n = 1). The ER group exhibited considerably lower late mortality rates than the BMT group (*P* = 0.004; Table 2).

### Aortic-related mortality

Aortic-related mortality was observed in 3 patients (1.8%) in the ER group and as many as 18 individuals (12.2%) in the BMT group (*P *= 0.001; Table 2).

### Survival

**Figure 2:**
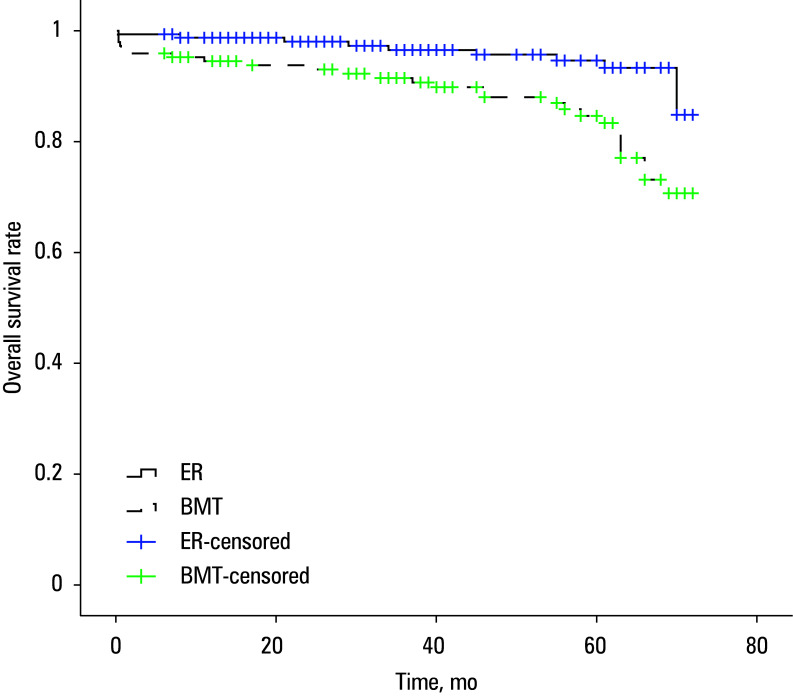
Overall survival rate of the groups (log-rank test; *P* = 0.001)

Abbreviations: see Figure 1

**Figure 3:**
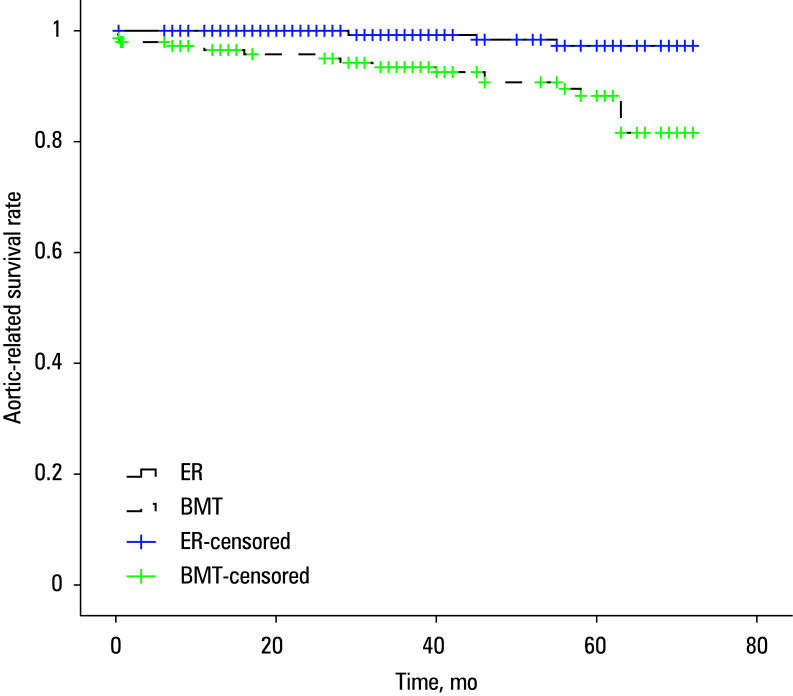
Aortic-related survival rate of the groups (log-rank test; *P* <⁠0.001)

Abbreviations: see Figure 1

Median (IQR) OS was 69 (68–71) months for the ER group and 64 (61–67) months for the BMT group (*P* = 0.001; Figure 2). In the ER group, the 1-, 3-, and 5-year OS rates were 98.8%, 96.5%, and 94.6%, respectively. Correspondingly, the BMT group exhibited OS rates of 94.5%, 91.5%, and 84.7%, respectiively, at 1, 3, and 5 years. Regarding aortic-specific survival, the ER group had 1-, 3-, and 5-year survival rates of 100%, 99.2%, and 97.3%, respectively, whereas the BMT group recorded corresponding rates of 96.5%, 93.4%, and 88.3% Figure 3.

## DISCUSSION

Our study compared the clinical outcomes associated with ER and BMT in patients with uncomplicated TBAD, evaluating efficacy of each method through thrombosed / obliterated false lumen rate, complications, mortality, and survival.

The primary goal of TBAD treatment is to prevent false lumen rupture and promote favorable false lumen remodeling.^5^;^6^;^7^ Our findings demonstrated that ER significantly improved the thrombosed / obliterated false lumen rate, as compared with BMT (81.8% vs 16.2%; *P* = 0.001). False lumen thrombosis is a strong predictor of reduced rupture risk and successful remodeling.^1^ Stent graft effectively seals the false lumen rupture and prevents blood flow into the false lumen, thereby promoting thrombosis.^1^ Additionally, stent graft placement increases true lumen diameter, decreases false lumen diameter, and achieves a 90% complete false lumen thrombosis rate over 5 years.^8^ Once false lumen thrombosis occurs, the rupture risk is markedly reduced. Our study also determined that ER resulted in a significant lowering of rupture rates in comparison with BMT (1.8% vs 12.2%; *P *= 0.001).

RTAD and organ failure are rare TBAD complications, with reported incidence rates below 5%.^1^ In the present study, the occurrence of both complications was comparable between the 2 groups, aligning with previous research.^4^;^9^;^10^ Type I endoleak, a complication specific to ER, occurred in 6% of the patients. However, this condition typically does not require intervention.^11^

While ER did not demonstrate a significant advantage over BMT in early mortality, it was associated with markedly lower late and aortic-related mortality. Among TBAD patients, false lumen rupture is the leading cause of death. Since ER prevents rupture, it contributes to a lower overall mortality rate.

Rates of 5-year OS in the ER and BMT groups (94.6% and 84.7%, respectively) were comparable to previously reported rates (90.8%–91.9% for ER and 82.2%–87.9% for BMT).^4^;^10^ Similarly, the 5-year aortic-specific survival rates (97.3% for ER and 88.3% for BMT) were consistent with previous findings (94.1%–100% for ER and 83.1%–86.1% for BMT).^8^;^10^

Stroke is a complication that rarely occurs following ER in TBAD patients, with an incidence of approximately 1.1%.^1^ In our study, no patients experienced stroke, likely due to the appropriate use of the chimney technique.

This study has several limitations. First, its retrospective nature introduces potential selection bias. Second, there was an imbalance in sample sizes between the 2 groups. However, the baseline characteristics of both groups were comparable, which may have mitigated bias. Third, as this study was conducted at a single center, further prospective, multicenter, randomized controlled trials are necessary to validate its findings.

## CONCLUSIONS

In comparison with BMT, ER can significantly reduce rupture rates and improve the thrombosed / obliterated false lumen rates, potentially leading to better long-term outcomes.
